# Lymphocyte-to-C-Reactive Protein Ratio as an Early Sepsis Biomarker for Neonates with Suspected Sepsis

**DOI:** 10.1155/2023/9077787

**Published:** 2023-05-08

**Authors:** Xiaojuan Li, Yulei Wei, Zhe Xu, Tiewei Li, Geng Dong, Xinrui Liu, Zhiwei Zhu, Jianwei Yang, Junmei Yang

**Affiliations:** ^1^Zhengzhou Key Laboratory of Children's Infection and Immunity, Children's Hospital Affiliated to Zhengzhou University, Henan Children's Hospital, Zhengzhou Children's Hospital, Zhengzhou, China; ^2^The Center of Henan Children's Neurodevelopmental Engineering Research, Children's Hospital Affiliated to Zhengzhou University, Henan Children's Hospital, Zhengzhou Children's Hospital, Zhengzhou, China

## Abstract

**Background:**

Neonatal sepsis is an extremely dangerous and fatal disease among neonates, and its timely diagnosis is critical to treatment. This research is aimed at evaluating the clinical significance of the lymphocyte-to-C-reactive protein ratio (LCR) as an early sepsis indicator in neonates with suspected sepsis.

**Methods:**

Between January 2016 and December 2021, 1269 neonates suspected of developing sepsis were included in this research. Among them, sepsis was diagnosed in 819 neonates, with 448 severe cases, as per the International Pediatric Sepsis Consensus. Data related to clinical and laboratory tests were obtained via electronic medical records. LCR was calculated as total lymphocyte (109 cells/L)/C-reactive protein (mg/L). Multivariate logistic regression analysis was employed to evaluate the effectiveness of LCR as an independent indicator for determining sepsis in susceptible sepsis neonates. Receiver operating characteristic (ROC) curve analysis was conducted for investigating the diagnostic significance of LCR in sepsis. When suitable, the statistical tool SPSS 24.0 was used for statistical analyses.

**Results:**

LCR decreased significantly in the control, mild, and severe sepsis groups. Further analyses exhibited that there was a substantially greater incidence of sepsis in neonates in the low-LCR group (LCR ≤ 3.94) as opposed to the higher LCR group (LCR > 3.94) (77.6% vs. 51.4%, *p* < 0.001). Correlation analysis indicated a substantial negative association of LCR with procalcitonin (*r* = −0.519, *p* < 0.001) and hospital stay duration (*r* = −0.258, *p* < 0.001). Multiple logistic regression analysis depicted LCR as an independent indicator for identifying sepsis and severe cases of this disease. ROC curve analysis indicated the optimal cutoff value of LCR in identifying sepsis to be 2.10, with 88% sensitivity and 55% specificity.

**Conclusions:**

LCR has proven to be a potentially strong biomarker capable of identifying sepsis in a timely manner in neonates suspected to have the disease.

## 1. Introduction

Compared with children and adults, neonates are more susceptible to pathogenic infections as their immune system is still developing. These infections may develop into sepsis [1]. The health and lives of neonates around the world are significantly threatened by neonatal sepsis, a systemic inflammatory response syndrome (SIRS) characterized by pathological inflammatory responses and organ system dysfunction [2]. Fleischmann-Struzek et al. [3] described that neonatal sepsis was estimated to affect 2202 out of every 100,000 live births, with a death rate of 11% to 19%. As a result, prompt and accurate diagnosis of neonatal sepsis is crucial for effective care. Currently, blood culture is used to diagnose neonatal sepsis [4]. However, the accuracy of blood culture depends on certain factors, such as blood volume, level of bacteremia, and prenatal and prehospital antibiotic use, which may result in a low positive rate of the blood culture [5]. In addition, the clinical presentation of sepsis is similar to other common neonatal diseases. Hence, finding novel biomarkers for neonatal sepsis is crucial.

In sepsis, multiple anti-inflammatory cytokines are released into the blood, further causing immunosuppression and subsequently leading to the apoptosis of many lymphocytes [6–8]. Several studies reported that lymphocytopenia was frequently observed in sepsis-affected individuals and was linked to poor outcomes [9–12]. When the body is inflamed or infected, the liver produces more C-reactive protein (CRP), an acute inflammatory protein [13]. Research has shown that CRP is a significant indicator of sepsis risk and a predictor in neonates and adults [14–17]. The lymphocyte-to-C-reactive protein ratio (LCR) is an index measured as the total lymphocyte count (TLC) level divided by the CRP level. Sepsis decreases lymphocyte counts and increases the CRP level [18, 19]. Therefore, this study proposed that LCR might be decreased in sepsis-affected neonates and thus can serve as a biomarker for identifying neonatal sepsis. Nevertheless, only a few studies have assessed the significance of LCR for this purpose. Thus, the primary goal of this research was to examine the clinical involvement of LCR in detecting neonatal sepsis.

## 2. Materials and Methods

### 2.1. Study Design and Population

This was a retrospective, single-center observational research that was carried out at the Henan Children's Hospital, Zhengzhou, China, between January 2016 and December 2021. Inclusion criteria for the study were neonates with suspected sepsis upon admission whose age is ≤28 days. Exclusion criteria were patients with a compromised immune system (liver failure, autoimmune diseases, and hematological cancer), cancer patients, or individuals suffering from severe congenital diseases. The study protocol was approved by the Hospital Ethics Review Board of the Henan Children's Hospital (no. 2022-K-105). Every research technique employed in this study was regarded as part of regular clinical practice, and the information was kept anonymous. Thus, considering the retrospective nature of the current investigation, informed consent was not required.

### 2.2. Clinical Definition

Neonates with suspected sepsis generally have one or more of the conditions mentioned as follows: respiratory distress, poor feeding, unstable body temperature, bradycardia, and abnormal white blood cells. The term “neonatal sepsis” refers to a systemic inflammatory response syndrome that includes a possible or confirmed infection. Severe sepsis can be described as sepsis combined with one of the following conditions: cardiovascular organ dysfunction, acute respiratory distress syndrome, or two or more other organ dysfunctions. As described by the published International Pediatric Sepsis Consensus, both neonatal sepsis and severe sepsis were diagnosed by two independent clinicians [20]. The control group included neonates with suspected sepsis who were eventually ruled out as they did not develop sepsis.

### 2.3. Data Collection

Data (demographics and laboratory tests) regarding patient age, gender, weight, body temperature, respiratory and heart rates, systolic and diastolic blood pressures, hospital stay duration, and procalcitonin (PCT), C-reactive protein (CRP), alanine aminotransferase (ALT), aspartate aminotransferase (AST), and albumin (ALB) levels were obtained via electronic medical records at the time of admission. Serum PCT concentration was calculated by employing the Cobas® 8000 modular analyzer (Roche Diagnostics, Switzerland). A latex-enhanced immunoturbidimetric assay on an UPPER analyzer (Ultrasensitive CRP kit, Upper Biotech, China) was utilized for the measurement of CRP levels. On an automatic Beckman biochemical analyzer (Beckman Coulter, California), the conventional clinical analytical approach was used to measure ALT, AST, and ALB levels. An automated blood cell counter (Sysmex Corporation, Japan) was employed to measure the white blood cell, neutrophil, and lymphocyte counts. In this study, CRP levels < 0.8 mg/L were measured to be 0.7 mg/L. PCT levels > 100 ng/mL or <0.02 ng/mL were measured as 101 ng/mL and 0.01 ng/mL, respectively. LCR was TLC (10^9^ cells/L)/CRP (mg/L).

### 2.4. Statistical Analysis

Independent *t*-tests or a one-way analysis of variance (ANOVA) was utilized for the assessment of normally distributed variables, which were reported as the mean ± standard deviation (SD). Non-normally distributed variables were expressed as medians (interquartile range) and assessed by employing the Mann-Whitney *U* test. Categorical variables were presented as percentages and evaluated via chi-square tests. Spearman's correlation test was employed to evaluate the association of LCR with other continuous variables. To determine the independent risk factor for the occurrence of severe sepsis and neonatal sepsis, multiple logistic regression analysis was applied, and it included variables having a *p* value < 0.05 in the univariate logistic analysis. Receiver operating characteristic (ROC) curve analysis was carried out for evaluation of the diagnostic significance of LCR in identifying neonatal sepsis. A comparison of the area under ROC curves (AUC) between two variables was done with the help of DeLong's test. The optimal cutoff point of LCR for identifying sepsis in neonates was calculated via Youden's index (sensitivity + specificity − 1) [21]. The SPSS version 24.0 (USA) and MedCalc version 15.2.2 (MedCalc Software, Belgium) were employed for analyzing all the data. A two-sided *p* value < 0.05 was deemed statistically significant.

## 3. Results

### 3.1. Study Population Characteristics

This research involved 1269 neonates with suspected sepsis. Among those neonates, 819 (64.5%) neonates were diagnosed with sepsis, of which, 371 (45.3%) were diagnosed as mild and 448 (54.7%) as severe. The remaining 450 (35.5%) neonates who did not develop sepsis were included in the control group. The baseline neonatal characteristics are summarized in Table 1. Unlike the control group, sepsis-affected neonates were older; had higher body weight, body temperature, and respiratory and heart rates; and had a lengthier hospital stay. Biochemical analysis and white blood cell count depicted that sepsis-affected neonates had increased PCT, CRP, ALT, and neutrophil count and lowered levels of ALB, lymphocyte count, and LCR. Furthermore, the differences in the above-related indexes among all three study groups were also analyzed. The PCT and CRP levels and the hospital stay duration exhibited a steady rise, and ALB and LCR levels gradually declined in all three study groups, according to the findings.

### 3.2. Association of LCR with Clinical Parameters

For the purpose of further evaluating the association of LCR with other clinical parameters, Spearman correlation analysis was conducted. As depicted in Table 2, LCR had a positive link to age (*r* = 0.126, *p* < 0.001), weight (*r* = 0.065, *p* = 0.021), ALB (*r* = 0.350, *p* < 0.001), and WBC (*r* = 0.230, *p* < 0.001) and a negative link to the body temperature (*r* = −0.126, *p* < 0.001), respiratory rate (*r* = −0.134, *p* < 0.001), heart rate (*r* = −0.094, *p* = 0.001), PCT (*r* = −0.519, *p* < 0.001), and neutrophil count (*r* = −0.101, *p* < 0.001). Moreover, a positive association of LCR was observed with hospital stay duration (*r* = −0.258, *p* < 0.001).

### 3.3. Independence of LCR in Identifying Neonatal Sepsis

Univariate and multivariable logistic analyses were carried out for assessing the clinical significance of LCR in predicting sepsis in neonates with suspected cases. The univariate logistic analysis showed that age, temperature, heart and respiratory rates, body weight, PCT, total neutrophil count, AST, ALT, and LCR were possible predictors for neonatal sepsis. After adjusting these statistically significant predictors of the univariate analysis, LCR was still an independent biomarker for neonatal sepsis (OR = 0.861, 95% CI 0.824–0.899, *p* < 0.001) and severe sepsis (OR = 0.936, 95% CI 0.898–0.975, *p* < 0.001). The data exhibited a negative and independent association of LCR with sepsis in neonates (Table 3).

### 3.4. Diagnostic Significance of LCR in Neonatal Sepsis

**Figure 1:**
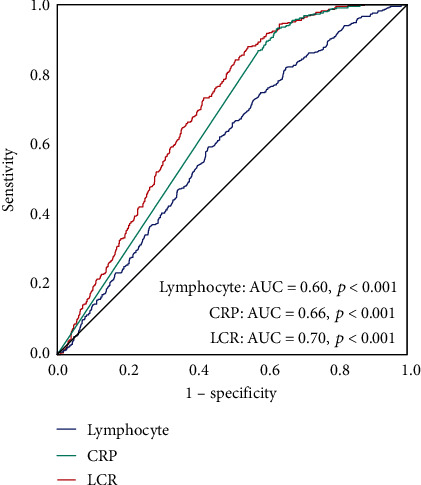
ROC curve of lymphocyte count, CRP and LCR in identifying neonatal sepsis.

**Figure 2:**
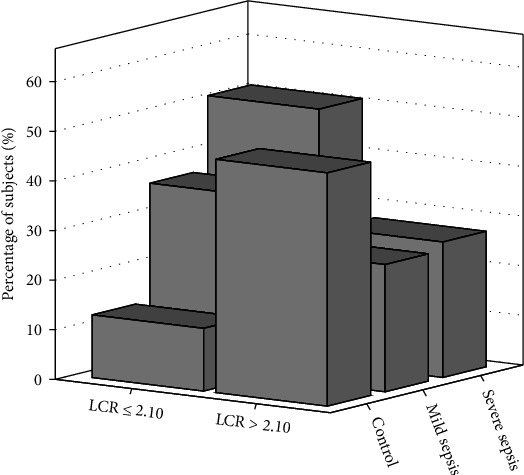
Division of neonates into high- and low-LCR groups.

ROC curve analysis was conducted for assessing the diagnostic significance of LCR in identifying sepsis in neonates. As depicted in Figure 1, the AUC for LCR in identifying neonatal sepsis was 0.70 (95% CI, 0.67–0.73, *p* < 0.001), which was higher than the AUC for lymphocyte count (AUC = 0.60, 95% CI, 0.57–0.63, *p* < 0.001) and CRP (AUC = 0.66, 95% CI, 0.63–0.69, *p* < 0.001) (*p* < 0.05). The optimal cutoff value of LCR in identifying neonatal sepsis was 2.10, with 88% sensitivity and 55% specificity. As per the cutoff value of LCR, two groups of neonates were established: the low-LCR group (LCR ≤ 2.10) and the high-LCR group (LCR > 2.10). As depicted in Table 4, there were 373 (87.4%) sepsis-affected neonates in the low-LCR group and 446 (53.0%) neonates without sepsis. The percentage of sepsis-affected neonates in the low-LCR group was substantially greater as opposed to that in the high-LCR group (Figure 2). Furthermore, analytic findings indicated the percentage of mild and severe sepsis-affected individuals to be elevated in the low-LCR group as opposed to the other group (Figure 2).

## 4. Discussion

Neonatal sepsis, also described as systemic inflammatory response syndrome, is a highly morbid and fatal condition with a bacterial, viral, or fungal origin characterized by hemodynamic alterations and clinical symptoms [22]. In a study involving 194 countries conducted between 2000 and 2013, Oza et al. [23] reported that neonatal sepsis accounted for the third highest number of neonate deaths following preterm birth and intrapartum complications, accounting for 15.6% of neonate fatalities. During the late neonatal period (7–27 days), the sepsis-associated death rate rose to 37.2% [23]. Accurate and timely diagnosis of sepsis in neonates can improve the clinical treatment of the condition and lessen antibiotic misuse. Currently available diagnostic techniques for neonatal sepsis depend on conventional blood culture, which is inefficient. Its findings may be affected by multiple factors, such as insufficient blood samples, administration of antibiotics prior to sampling, reduced bacteria in the blood, or short-term bacteremia. Moreover, sepsis symptoms in neonates are quite generalized [24]. Therefore, identifying new biomarkers of neonatal sepsis is important.

Lymphocytes are a type of white blood cell produced in the bone marrow. In the body, these cells fight off bacterial and viral infections. However, with sepsis, there is extensive apoptotic death of lymphocytes [8]. An essential stage in the onset of experimental sepsis has been identified as lymphocyte apoptosis. This process can further induce a state of immunosuppression, which increases host susceptibility to invading pathogens [8, 25, 26]. Meanwhile, several studies reported that peripheral blood lymphocyte count decreased in patients with sepsis, and lymphopenia was associated with poor outcomes [10, 27–29]. CRP was one of the most studied and frequently used inflammation markers. As a traditional inflammatory marker, CRP has proven its potential as an independent sepsis risk factor [15, 16, 30]. However, Pradhan et al. [31] found that CRP is a sensitive marker of sepsis, but not specific. For neonatal sepsis, the sensitivities and specificities of CRP in diagnosing sepsis ranged from 29% to 100% and from 6% to 100%, respectively [32]. During this research, the data also exhibited a high sensitivity (93%) and a low specificity (37%) of CRP in diagnosing neonatal sepsis (data not shown).

Recently, as an emerging marker of systemic inflammation, LCR has received increasing attention. Particularly in cancer, this ratio is more sensitive during the acute inflammatory phase. Studies have demonstrated that LCR is a safe and effective postsurgical prognostic biomarker of postoperative complications in patients with gastric cancer, esophageal cancer, colorectal cancer, and hepatocellular carcinoma [33–37]. In terms of infectious diseases, recent studies found that LCR was closely related to the coronavirus disease 2019 (COVID-19) [38, 39]. Yang et al. [40] reported that LCR proved more beneficial as opposed to CRP or lymphocytes alone in the assessment of severe COVID-19. In the timely identification and anticipation of the severity and fatality of COVID-19, the CRP-to-lymphocyte ratio (CLR) may be a helpful prognostic indicator. However, the relationship of LCR with sepsis in adults and neonates has received little attention from published research.

During this research, the association of LCR with neonatal sepsis was initially studied. It was observed that sepsis-affected neonates had reduced levels of LCR, and these values were reduced gradually within all three study groups. Multivariate analysis exhibited the potential of LCR as an independent predictor in distinguishing septic neonates from neonates with suspected sepsis. ROC curve analysis depicted the better discriminatory capability of LCR in contrast with lymphocyte count and CRP in identifying neonatal sepsis. The optimal cutoff value of LCR in diagnosing sepsis among neonates was 2.10, with 88% sensitivity and 55% specificity. Using the optimal cutoff value, the neonates were further stratified into two groups, and it was observed that the low-LCR group exhibited an increased occurrence of neonatal sepsis (87.4%) and had a low percentage of controls (12.6%).

There are several limitations to this research. Because this is a retrospective single-center observational study, additional confounding factors could affect the results no matter how many exclusion criteria are used. In addition, multicenter clinical investigations are required in the future to confirm the findings. All neonates with a final diagnosis of sepsis were clinical sepsis. As a result, it is possible that the actual incidence rate of neonatal sepsis is overestimated or underestimated. Additionally, LCR was only calculated when patients were admitted to the hospital, and serial measurements of LCR might offer more effective monitoring of the relationship between LCR and neonatal sepsis.

## 5. Conclusions

The present study has proven that LCR is a useful biomarker in distinguishing septic neonates from neonates with suspected sepsis. These findings highlight that LCR, which can be measured and calculated easily, quickly, and cost-effectively, can serve as a promising alternative way of efficiently identifying neonatal sepsis.

## Figures and Tables

**Table 1:** Basic characteristics of study subjects by groups.

Variables	Control (*n* = 450)	Sepsis (*n* = 819)	*p* ^∗^	Sepsis		*p* ^#^
				Mild sepsis (*n* = 371)	Severe sepsis (*n* = 448)	
Age (days)	7.0 (4.0, 12.0)	10.0 (5.0, 17.0)	<0.001	5.0 11.0, 18.0)	10.0 (5.0, 16.0)	0.305
Male, *n* (%)	252 (56.0%)	497 (60.7%)	0.105	222 (59.8%)	275 (6.4%)	0.242
Weight (kg)	3.29 ± 0.51	3.45 ± 0.65	0.027	3.35 ± 0.57	3.11 ± 0.70	<0.001
Temperature (°C)	36.98 ± 0.46	37.36 ± 0.76	<0.001	37.38 ± 0.71	37.33 ± 0.80	0.429
Respiratory (rate/minute)	46.60 ± 8.00	50.25 ± 11.93	<0.001	49.81 ± 9.96	50.62 ± 13.35	0.336
Heart rate (bpm)	142.24 ± 16.47	149.84 ± 19.61	<0.001	148.34 ± 18.82	151.08 ± 20.18	0.047
SBP (mm, Hg)	76.24 ± 6.81	75.93 ± 8.49	0.508	78.43 ± 6.54	73.86 ± 9.33	<0.001
DBP (mm, Hg)	46.43 ± 7.00	45.96 ± 8.00	0.289	47.39 ± 7.49	44.77 ± 8.22	<0.001
PCT (ng/mL)	0.14 (0.10, 0.25)	0.32 (0.14, 1.53)	<0.001	0.23 (0.11, 0.81)	0.43 (0.16, 2.39)	<0.001
CRP (mg/L)	0.7 (0.7, 0.7)	0.7 (0.7, 15.3)	<0.001	0.7 (0.7, 11.1)	0.7 (0.7, 17.7)	0.064
ALB (g/L)	33.76 ± 4.07	30.48 ± 4.96	<0.001	31.49 ± 4.95	29.64 ± 4.92	<0.001
ALT (U/L)	25.7 (20.1, 33.5)	28.2 (21.3, 37.4)	<0.001	28.1 (21.1, 35.8)	28.5 (21.6, 39.9)	0.088
AST (U/L)	38.1 (30.3, 51.3)	37.7 (27.7, 54.5)	0.372	36.1 (27.4, 48.6)	39.2 (28.2, 63.3)	0.003
WBC (×10^9^ cells/L)	10.03 (8.09, 12.54)	10.13 (7.39, 14.65)	0.370	9.84 (7.66, 13.37)	10.52 (6.96, 15.72)	0.244
Neutrophil (×10^9^ cells/L)	4.24 (3.17, 6.25)	5.30 (3.16, 8.71)	<0.001	4.80 (3.21, 8.11)	5.70 (3.13, 9.52)	0.091
Lymphocyte (×10^9^ cells/L)	3.98 (2.95, 5.25)	3.39 (2.10, 4.75)	<0.001	3.47 (2.34, 4.62)	3.30 (1.87, 4.90)	0.157
LCR	5.34 (3.38, 7.25)	2.61 (0.20, 5.71)	<0.001	3.32 (0.24, 6.04)	2.26 (0.14, 5.30)	0.031
Length of hospital stay (days)	9.0 (8.0, 11.0)	15.0 (10.0, 23.0)	<0.001	13.0 (9.0, 18.0)	18.0 (12.0, 26.0)	<0.001

Notes: all values are presented as the mean ± SD or *n* (%) or as the median (interquartile range). ^∗^*p* value among the control and sepsis groups. ^#^*p* value among the control, mild, and severe sepsis groups. Abbreviations: SBP: systolic blood pressure; DBP: diastolic blood pressure; PCT: procalcitonin; CRP: C-reactive protein; ALB: albumin; ALT: alanine aminotransferase; AST: aspartate aminotransferase; WBC: white blood cell; LCR: lymphocyte-to-C-reactive protein ratio.

**Table 2:** Correlations between LCR and clinical parameters.

Variables	*r*	*p*
Age (day)	0.126	<0.001
Temperature (°C)	-0.126	<0.001
Respiratory (rate/minute)	-0.134	<0.001
Heart rate (bpm)	-0.094	0.001
Weight (kg)	0.065	0.021
SBP (mm, Hg)	0.055	0.052
DBP (mm, Hg)	0.029	0.300
PCT (ng/mL)	-0.519	<0.001
ALB (g/L)	0.350	<0.001
ALT (U/L)	-0.017	0.537
AST (U/L)	-0.023	0.423
WBC (×10^9^ cells/L)	0.230	<0.001
Neutrophil (×10^9^ cells/L)	-0.101	<0.001
Length of hospital stay (days)	-0.258	<0.001

Abbreviations: SBP: systolic blood pressure; DBP: diastolic blood pressure; PCT: procalcitonin; CRP: C-reactive protein; ALB: albumin; ALT: alanine aminotransferase; AST: aspartate aminotransferase; WBC: white blood cell.

**Table 3:** Regression analysis to assess the presence of neonatal sepsis and severe sepsis according to LCR.

Variables	Univariate		Multivariate^∗^	
	OR (95% CI)	*p*	OR (95% CI)	*p*
Presence of total sepsis				
LCR	0.835 (0.805–0.867)	<0.001	0.861 (0.824–0.899)	<0.001
LCR group				
Low LCR group	1		1	
High LCR group	0.305 (0.239–0.389)	<0.001	0.389 (0.292–0.519)	<0.001
Presence of severe sepsis				
LCR	0.883 (0.850–0.916)	<0.001	0.936 (0.898–0.975)	0.002
LCR group				
Low LCR group	1		1	
High LCR group	0.431 (0.340–0.547)	<0.001	0.601 (0.459–0.788)	<0.001

Notes: ^∗^adjusted for age, temperature, heart rate, respiratory rate, body weight, PCT, total neutrophil count, AST, and ALT. Abbreviation: LCR: lymphocyte-to-C-reactive protein ratio.

**Table 4:** Distribution of neonates with/without sepsis based on the optimal cutoff point of LCR.

Variables	LCR ≤ 2.10 (*n* = 427)	LCR > 2.10 (*n* = 842)	*p*
Control, *n* (%)	54 (12.6%)	396 (47.0%)	<0.001
Total sepsis, *n* (%)	373 (87.4%)	446 (53.0%)	<0.001
Mild sepsis, *n* (%)	209 (32.9%)	216 (25.7%)	0.004
Severe sepsis, *n* (%)	284 (44.7%)	230 (27.3%)	<0.001

Abbreviation: LCR: lymphocyte-to-C-reactive protein ratio.

## Data Availability

The data used to support the findings of this study are available from the corresponding author upon request.

## Abbreviations

SBP: Systolic blood pressure
DBP: Diastolic blood pressure
PCT: Procalcitonin
CRP: C-reactive protein
ALB: Albumin
ALT: Alanine aminotransferase
AST: Aspartate aminotransferase
WBC: White blood cell
LCR: Lymphocyte-to-C-reactive protein ratio.
